# Sjögren's Syndrome Complicated by Myeloid/Natural Killer Cell Precursor Acute Leukemia: Case Report and Review of the Literature

**DOI:** 10.1155/2016/8261249

**Published:** 2016-09-25

**Authors:** Hao Feng, Jianlin Qiao, Ningning Ding, Wei Chen, Kunming Qi, Xiuying Pan, Jiang Cao, Kailin Xu

**Affiliations:** Department of Hematology, The Affiliated Hospital of Xuzhou Medical University, Jiangsu, China

## Abstract

We report a case of Sjögren's syndrome (SS) complicated by myeloid/natural killer (NK) cell precursor acute leukemia (M/NKPAL). A 75-year-old woman with a previous SS history for 2 years was routinely treated. Peripheral blood progenitor cells were increased, and subsequent bone marrow cell morphology examination showed the presence of acute myeloid leukemia type M4. However, flow cytometry analysis revealed that CD7/CD56/CD33/CD34/HLA-DR/cCD3 were all positive and myeloperoxidase- (MPO-) specific staining, other T cells, NK cells, and myeloid markers were all negative. Clonal T-cell receptor (TCR)*β*/TCR*γ*/TCR*δ* gene rearrangements and Epstein-Barr virus (EBV) were negative. The diagnosis of M/NKPAL was therefore confirmed. Unfortunately, this patient did not receive chemotherapy and later died of acute left heart failure and respiratory failure. SS complication with M/NKPAL is relatively rare. Combined with the relevant literatures, our study offers new insights into the clinical characteristics, pathological features, possible pathogenesis, and differential diagnosis of this rare disease.

## 1. Introduction

Autoimmune diseases have been associated with an increased risk of haematological malignancies [1]. Sjögren's syndrome (SS) is a chronic inflammatory autoimmune disease primarily involving the exocrine glands. SS can be concurrent with second tumors, mostly non-Hodgkin's lymphoma (NHL) [1, 2] and occasionally T-cell leukemia/lymphoma [3]. However, SS concurrent with natural killer (NK) cell leukemia/lymphoma is very rare, and SS concurrent with myeloid/NK cell precursor acute leukemia (M/NKPAL) has not been reported.

## 2. Case Presentation

**Figure 1:**
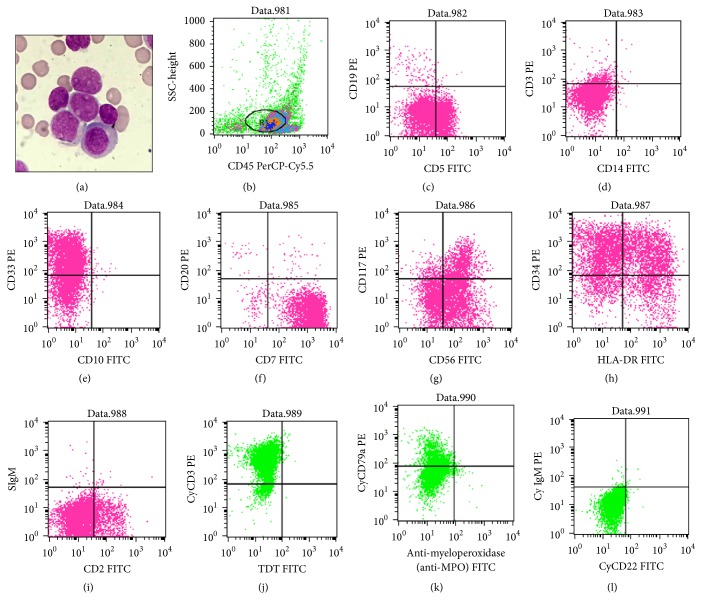
(a) May-Giemsa staining of bone marrow smear (magnification ×1000); (b–i) histogram of flow cytometry of bone marrow specimens (BD Bioscience, San Jose, CA, USA).

We present the case of a 75-year-old female patient who had a 2-year history of SS and had routinely taken methylprednisolone and hydroxychloroquine sulphate tablets. She was admitted to hospital in December 2014 because the peripheral blood blast cells increased for 2 days. Peripheral blood test results are shown in Table 1. Furthermore, when peripheral blood smear was detected, 12% blast cells were found. Bone marrow cell morphology examination revealed a myeloid hyperplasia with 44% leukemic cells and large cell bodies with various sizes (Figure 1(a)). The nuclei of the cells were round and oval-shaped, with positional deviation. The chromatin appeared fine and flat with rare nucleoli and the cytoplasm was abundant, grey-blue with no visible particles. Some of the cells showed visible protrusions and vacuoles. The number of lymphocytes decreased, and mononuclear cells significantly decreased, with a proportion of 30% immature mononuclear cells. Erythroid and megakaryocytic populations were decreased, and leukocyte peroxidase (POX) staining was negative. These features constituted the manifestation of type M4 acute myeloid leukemia (AML). Bone marrow flow cytometry (BD Bioscience, San Jose, CA, USA) analysis revealed that CD7/CD56/CD34/CD33/HLA-DR/cyCD3 markers were positive. Myeloperoxidase- (MPO-) specific staining was negative. Other T cells, NK cells, and myeloid markers were all negative, which supported the diagnosis of M/NKPAL (Figures 1(b)–1(i)). In addition, Epstein-Barr virus (EBV) detection and clonal T-cell receptor (TCR)*β*/TCR*γ*/TCR*δ* gene rearrangements were negative, which further supported the diagnosis. The bone marrow chromosome examination yielded 46, XX.

The patient exhibited a poor mental state with mild anaemia. Routine blood test showed anaemia and low platelet counts but no clinical manifestation of bleeding. Multiple oral ulcers had occurred. The tongue body exhibited hypertrophy accompanied with gingival hyperplasia. Multiple superficial lymph node enlargements were observed, with the left armpit node being the largest, approximately 1.5 × 1.1 cm, soft with no obvious tenderness. Sternal tenderness was obvious, but no swelling of the liver and spleen under the ribs was observed. Chest computed tomography (CT) revealed pneumonia. Because the patient refused a low-dose chemotherapy regimen, only methylprednisolone for SS was applied, along with supportive therapy, including mezlocillin plus sulbactam (anti-infection) and atomization inhalation.

Recurrent fever was observed after patient admission, with the highest being 39.2°C. A fungal 1,3-*β*-D glucan + galactomannan (G + GM) assay showed a fungal infection, and voriconazole was used as an antifungal treatment. On the thirteenth day after admission, acute left heart failure occurred, and the patient was transferred to the intensive care unit (ICU). After proactive treatment, the patient's symptoms still showed no improvement. The patient died on the fifteenth day due to acute left heart failure, respiratory failure, and invalid rescue.

## 3. Discussion

SS is a chronic autoimmune disease primarily involving exocrine glands throughout the entire body. Its symptoms are mainly manifested at the salivary and lacrimal glands. Other systems, such as respiratory system, digestive system, and skin external glands, might also be affected. The main pathological damage includes lymphocytic infiltrations, such as an emerging number of lymphocytes between exocrine glands composed of columnar epithelial cells, the infiltration of plasma cells and mononuclear cells, and the formation of lymphoid follicle-like structures. SS complication with other tumours has been reported [4], but related causes and mechanisms are still unclear. SS may be a complex process involving multiple factors. Masaki and Sugai [5] suggested that SS from the infiltration of lymphocytes resulting in lymphoma was a multistage process: firstly, polyclonal lymphocyte proliferation is replaced with monoclonal proliferation, which further develops into mucosa-associated lymphoid tissue lymphoma (MALT) and eventually becomes an aggressive malignant lymphoma. In addition, lymphoepithelial sialadenitis (LESA) is essential for the occurrence of MALT at salivary lymphatic glands [2, 6]. LESA may be associated with monoclonal B-cell proliferation and cytokine dysregulation, such as the expression of monoclonal rheumatoid factor and type I and type II interferons (IFNs) [7, 8]. However, Lossos and Morgensztern [9] discovered that gene mutation, amplification, and deletion could all occur in the process of SS-concurrent NHL. The most common manifestation was ectopic recombination of chromosomes. For example, p53 is an important antioncogene that plays an important role in the tumour proliferation and transformation process. p53 has been suggested to play a regulatory role in SS progression to NHL; therefore, p53 mutation-induced dysregulation might be an important factor for determining if SS will develop into lymphoma [10]. Voulgarelis and Skopouli [2] analysed a possible mechanism of SS transformation into lymphoma: first, on the basis of the SS-induced lymphadenitis reaction, the selective inhibition of apoptosis factor antigens results in the amplification of monoclonal B cells. Then, when other carcinogenic effects, including p53 mutation and chromosome translocation, were in place, SS transforms into malignant lymphoma.

Yan et al. [11] reported an SS case complication with nasal NK/T cell lymphoma accompanied by positive antinuclear antibodies (ANA) and anti-SS-A/Ro antibody and rheumatoid factor (RF) and negative anti-SS-B/La antibody. CD56 and CD20 expression was negative, but CD3*ε*, granzyme B expression, and EB virus were positive. The patient in the present study was hospitalized with poorly controlled SS, and their RF, ANA, anti-SS-A/Ro, and anti-SS-B/La results and other indicators were beyond the normal ranges. The abnormal SS indices before the M/NKPAL complication indicated that SS may be related to the occurrence of M/NKPAL.

The incidence of M/NKPAL is very low, with only 30 cases reported [12, 13]. However, referring to the 2016 WHO classification, M/NKPAL is not included as one of “acute leukemias of ambiguous lineage” [14]. The first case was reported by Suzuki et al. in 1997 [15], with a diagnosis of M/NKPAL. The characteristics of M/NKPAL were as follows. (1) In the early stage, pancytopenia, fatigue, fever, pain, and skin ecchymosis were visible. Extramedullary infiltration, peripheral lymph node and mediastinal enlargement, and hepatosplenomegaly were also observable. (2) An EBV test was negative. (3) The leukemia cell morphology was similar to acute lymphoblastic leukemia (ALL-L2), lymphoblastoid cells, or mononuclear tumours. POX, PAS (periodic acid-Schiff stain), and esterase staining were negative. (4) Immunophenotype analysis revealed the expression of both myeloid and NK cell antigens, mainly CD7+/CD56+/CD34+/CD33+/CD3−. HLA-DR was mostly positive, cyMPO was partially positive, and other antigens of NK, B, and T cells were not expressed. TCR*β*/TCR*γ*/TCR*δ* gene rearrangements were mostly negative.

For the immunophenotype analysis of M/NKPAL, Suzuki and Nakamura [16] discovered that, among the main manifestations of M/NKPAL, CD7 is one of the most sensitive antigen indices to detect T cell acute lymphoblastic leukemia (T-ALL) but its specificity is not very strong. The authors proposed that CD7 antigen could also be expressed in mature and immature NK cells and myeloid stem cells, suggesting that CD7 expression is an indication of the high primeval degree of M/NKPAL cells. Therefore, CD7 may originate from T/NK progenitor cells with dual differentiation potential. CD56 antigen is often considered as an NK cell marker. In the classical differentiation pathway of NK cells, bone marrow CD34+ hematopoietic stem cells differentiate into myeloid progenitor cells and lymphoid progenitor cells. Lymphoid progenitor cells can then differentiate into B cells, T cells, dendritic cells, and NK cells [17, 18]. First, NK cell progenitor cells are derived from lymphoid hematopoietic progenitor cells with an immunophenotype of CD34+/CD33+/CD38+/CD7+. Subsequently, NK progenitor cells differentiate into NK CD7+/CD34+/CD33+/CD117+/CD56+/−/CD16− precursor NK progenitor cells. In the third stage, the NK progenitor cells differentiate into immature NK cells with a phenotype of CD34−/CD33+/CD56+/CD117+. The fourth and fifth stages represent mature NK cells. The difference between the stages is that the former group of NK cells mainly manifests in the lymph nodes and tonsils, and CD56 expression is strongly positive and CD16 is weakly positive or negative. The latter mature NK cells weakly express CD56 and strongly express CD16. NK cells further mature and distribute in the peripheral blood and the spleen, suggesting that CD7 and CD56 coexpression indicates that M/NKPAL derives from higher primitive stages of differentiation. In addition, CD16 is a marker of NK cells, whose expression becomes gradually positive during NK cell maturation process. The CD16-negative result also indicated that the disease's tumour cells were phenotypically immature [19].

Although the immunophenotype of cyCD3 was positive, the clinical characteristics and laboratory examination results of the patient in this study were essentially consistent with an observation on M/NKPAL. cyCD3 was regarded as a more specific marker for the T cell system. Early NK cell cytoplasms contained CD3*ε* mRNA and cyCD3 antigen and were downregulated with cell maturation and differentiation. Therefore, M/NKPAL cells could express cyCD3. However, cyCD3 should be positively expressed but weakly, and CD3 and TCR gene rearrangements should be negative [20].

In the past, B cells and related cytokines have been considered to play important roles in SS pathogenesis. Recent studies have suggested that the roles of T cells and NK cells in such disease should not be ignored [21]. Although the incidence of M/NKPAL is low, the disease has a high misdiagnosis rate, rapid progression, and poor prognosis. Some patients have even died before treatment initiated. However, with more reports regarding relevant cases and persistent research efforts on SS and M/NKPAL pathogenesis, we believe that the relationship between these two diseases will soon be understood in depth.

## Figures and Tables

**Table 1:** Patient's main laboratory features of peripheral blood.

	Values	References
White blood cells (10^9^/L)	4.3	3.5–9.5
Neutrophils%	5.3	51–75
Lymphocytes%	89.9	20–50
Haemoglobin (g/L)	97	115–150
Platelet (10^9^/L)	37	125–350
C-reactive protein (mg/L)	80.7	0–5
Antinuclear antibodies	8.7	<1
Double-stranded DNA antibodies (IU/mL)	67.9	<25
Anti-Ro antibodies (IU/mL)	>200	<25
Anti-La antibodies (IU/mL)	>200	<25 L
Rheumatoid factor (IU/mL)	30	0–25
